# Angiotensin II-Treated Cardiac Myocytes Regulate M1 Macrophage Polarization via Transferring Exosomal PVT1

**DOI:** 10.1155/2021/1994328

**Published:** 2021-08-30

**Authors:** Feng Cao, Zhe Li, Wenmao Ding, Ling Yan, Qingyan Zhao

**Affiliations:** ^1^Department of Cardiology, Renmin Hospital of Wuhan University, Wuhan 430060, China; ^2^Cardiovascular Research Institute, Wuhan University, Wuhan 430060, China; ^3^Hubei Key Laboratory of Cardiology, Wuhan 430060, China

## Abstract

Atrial fibrillation (AF) seriously reduces the health and life quality of patients. It is necessary to explore the pathogenesis of AF and provide a new target for the treatment. Here, exosomes were identified using transmission electron microscopy and nanoparticle tracing analysis. Western blotting assay was performed to detect the expression of exosomal surface markers, extracellular matrix-related proteins, and IL-16. The expression of genes was measured using qRT-PCR. Flow cytometry was performed to examine the percentages of CD86- and CD163-positive macrophages. Besides, luciferase activity assay was performed to explore the combination between PVT1 and miR-145-5p and the combination between miR-145-5p and IL-16 3'UTR. The combination between PVT1 and miR-145-5p also was examined using RIP assay. In our study, we isolated human cardiac myocyte- (HCM-) derived exosomes successfully. Ang-II-treated HCM-derived exosomes (Ang-II-Exo) promoted M1 macrophage polarization. PVT1 was highly expressed in Ang-II-Exo. Ang-II-Exo induced macrophage to M1 polarization through transferring PVT1. Furthermore, our data showed that PVT1 increased the expression of IL-16 via sponging miR-145-5p. Finally, we proved that exosomal PVT1 could boost the extracellular matrix remodeling of atrial fibroblasts. Overall, our data demonstrated that Ang-II-Exo promoted the extracellular matrix remodeling of atrial fibroblasts via inducing M1 macrophage polarization by transferring PVT1. PVT1 facilitated M1 polarization macrophage via increasing IL-16 expression by sponging miR-145-5p. Our results provided a new evidence for PVT1 which might be a treatment target of AF.

## 1. Introduction

Atrial fibrillation (AF) is the most common cardiac arrhythmia and the main reason for embolic stroke. AF has an unsatisfactory outcome and a high mortality in clinic. Besides, the occurrence and development of extracranial systemic thromboembolism, dementia, heart failure, myocardial infarction, and venous thromboembolism have also been proved to be closely associated with AF [1]. Currently, more than 33 million populations suffer from AF globally, and the incidence of AF is still increasing [2]. A better understanding of the pathogenesis of AF is very necessary for improving the therapeutic status of AF. Scientific evidence has demonstrated that atrial fibrosis is the main characteristic of cardiac structural remodeling in cardiovascular diseases, including AF [3]. However, the data about the regulatory mechanism of atrial fibrosis are limited. It was demonstrated that extracellular matrix (ECM) remodeling plays a crucial role in the development of cardiac diseases. Excessive matrix metalloproteinase 9 (MMP-9), collagen I, collagen III, and other ECM-related protein deposition is an important pathological characteristic of atrial fibrosis [4]. Therefore, exploring an effective method to inhibit ECM remodeling may provide a new hope for atrial fibrosis treatment.

Inflammatory cells play an important role in the process of atrial fibrosis. During the process, a large number of inflammatory cells (especially macrophages) rapidly infiltrate into the infarct area [5, 6]. In the infarct region, activated macrophages could facilitate the development of atrial fibrosis through communicating with other cells like cardiac myocytes and fibroblasts via transmitting chemokines and proinflammatory cytokines [7]. Furthermore, under different pathological conditions, macrophages can differentiate into two different phenotypes: proinflammatory M1 phenotype and anti-inflammatory M2 phenotype [8]. Sun et al. indicated that the percentage of M1-polarized macrophage is significantly increased in AF [9]. Importantly, a large number of researches have suggested that atrial fibrosis can be effectively promoted by M1 macrophage polarization, while it can be improved by M2 macrophage polarization [10]. In this study, we explored the relationships among cardiac myocytes, macrophage polarization, and ECM remodeling in atrial fibroblasts.

Long noncoding RNAs (lncRNAs) are a class of transcripts with more than 200 nucleotides in length. LncRNAs participate in the progression of multiple disorders [11]. Our previous study revealed that lncRNA PVT1 is increased in the atrial muscle tissues of the patients with AF. Upregulation of PVT1 could accelerate AF development [12]. Nevertheless, whether PVT1 affects the interaction between cardiac myocytes and macrophage remains unclear. Exosomes are a key mediator for the communication among different cells via submitting small molecules like lncRNA. Exosomes can be produced by almost all cell types. Recently, Zhao et al. proved that bone marrow mesenchymal stem cell-derived exosomal PVT1 could regulate the cell proliferation and metastasis of osteosarcoma cells via acting as a miR-183-5p sponge [13]. In addition, interleukin 16 (IL-16) is an immunomodulatory chemokine. It was demonstrated that IL-16 is increased in M1-polarized macrophages, and miR-145 could facilitate M2 macrophage polarization through suppressing IL-16 expression [14]. In this study, we proved that angiotensin II- (Ang-II-) induced cardiac myocyte-derived exosomal PVT1 boosted the ECM remodeling in atrial fibroblasts by promoting M1 macrophage polarization through targeting the miR-145-5p/IL-16 signaling pathway. Our data indicates a novel regulatory mechanism of atrial fibrosis. In the meantime, our data provide a new evidence for PVT1 acting as a potential treatment target of AF.

## 2. Materials and Methods

### 2.1. Cardiac Myocyte Culture and Treatment

Human cardiac myocytes (HCMs) were purchased from the ScienCell Research Laboratories (#6200, Carlsbad, California, USA). In this study, HCMs were cultured in the complete cell culture medium which was mixed with Dulbecco's Modified Eagle's Medium (DMEM; Mediatech, Herndon, VA, USA) and 10% fetal bovine serum (FBS; Gibco BRL, USA). All cells were cultured in an incubator with 5% CO_2_ and room air at 37°C. For the treatment of HCMs, the cells were treated with 1 *μ*M Ang-II or equal volume of PBS for 48 hours.

### 2.2. Exosome Isolation and Identification

Vesicles were collected from cell culture medium of the HCMs treated with PBS or Ang-II. Firstly, the supernatant was centrifuged at 2000 g for 20 min to remove cell fragments. Then, the supernatant was centrifuged at 10,000 g for 30 min. Next, the supernatant was centrifuged again at 100,000 g for 70 min. All centrifugation works were carried out at 4°C. After centrifugation, the final sediment was collected, and subsequently the vesicles were resuspended into 0.01 M PBS solution. Finally, the characteristic surface markers (Alix, HSP70, and CD63) of exosomes were identified using western blot and flow cytometry. Meanwhile, the morphology and diameter of vesicles were measured using transmission electron microscopy (TEM) and nanoparticle tracking analysis (NTA), respectively. Vesicles were fixed with 2.5% glutaraldehyde overnight at 4°C. On the next day, exosomes were loaded onto the formvar carbon-coated grids, and were stained with aqueous phosphotungstic acid for 1 min. At last, a TEM at 80 kV was used to analyze the morphology of exosomes.

### 2.3. Western Blotting Assay

Total protein was isolated from exosomes and cells using RIPA lysis buffer (Santa Cruz Biotechnology, Inc., Dallas, TX, USA). Then, equal quality of total protein (20 *μ*g) from each group was loaded into the 12% SDS-PAGE and was separated on it. Next, all proteins were transferred into a PVDF membrane. The PVDF membrane was then maintained with 5% nonfat milk for 1 hour at room temperature. After that, the membrane was incubated with the working solution of primary antibodies at 4°C overnight. The primary antibodies included anti-Alix (1 : 1000, Abcam, Cambridge, MA, USA), anti-HSP70 (1 : 2000, Abcam), anti-CD63 (1 : 1000, Abcam), anti-IL-16 (1 : 2000, Cell Signaling Technology, Inc., CST, Danvers, Massachusetts, USA), anti-MMP-9 (1 : 2000, Abcam), anti-tissue inhibitors of MMP-1 (TIMP-1) (1 : 1000, Abcam), anti-collagen I (1 : 1000, Abcam), anti-collagen III (1 : 1000, Abcam), and anti-*β*-actin (1 : 3000, CST). Subsequently, all membranes were incubated with the horseradish peroxidase-labeled secondary antibody for another 1 hour at room temperature. Finally, an enhanced chemiluminescence kit (Pierce Chemical Co., Rockford, IL, USA) was used to visualize the protein bands which were then analyzed using software Quantity One. Here, *β*-actin served as the loading control of other tested proteins.

### 2.4. Flow Cytometry Assay

Flow cytometry assay was carried out to analyze the percentages of CD63-positive vesicle, CD163-positive macrophage, and CD86-positive macrophage. Exosomes or macrophages were resuspended into PBS, and then were incubated with 5 *μ*l of FITC-labeled anti-CD63 (Becton Dickinson, New Jersey, USA), 5 *μ*l of FITC-labeled anti-CD86 (Becton Dickinson), or 5 *μ*l of PE-labeled anti-CD163 (Becton Dickinson) for 30 min at room temperature. Finally, the positive exosomes or cells were analyzed using a BD Accuri™ C6 Flow Cytometer (BD Biosciences).

### 2.5. Macrophage Culture and Induction

Human monocytic leukemia cell line THP-1 was obtained from Kunming Institution of Zoology (Yunnan, China). All cells were cultured in the complete culture medium at 37°C in an incubator with 5% CO_2_. For the induction of THP-1, the exosomes were obtained from HCMs treated with PBS or Ang-II. Then, THP-1 cells were incubated with 80 *μ*g/ml exosomes for 24 h to induce M1 or M2 macrophage polarization. Next, the polarization of macrophage was measured using western blot assay, flow cytometry assay, and qRT-PCR assay.

### 2.6. qRT-PCR Assay

Total RNA was isolated from exosomes using miRCURY™ RNA isolation kit (Exiqon, Vedbaek, Denmark) and was isolated from cells using TRIzol reagent (Invitrogen, Thermo Fisher Scientific, Waltham, MA, USA). Then, a Reverse Transcription System Kit (Takara, Dalian, China) was utilized to reverse transcribe RNA into cDNA. Next, qRT-PCR was carried out using the SYBR Green PCR Master Mix (Thermo Fisher Scientific) on a PRISM 7500 Real-Time PCR System (Applied Biosystems, Waltham, MA, USA). Finally, the relative expression levels of gene were analyzed according to the classical 2^-*ΔΔ*Ct^ method. Here, *U6* served as the internal reference of miR-145-5p. The relative expression levels of PVT1 and mRNAs were normalized to *GAPDH*. All experiments were accomplished according to the specific manufacturer's instruction. The primer sequences are listed in Supplementary Table 1.

### 2.7. Cell Transfection

The lentivirus expressing PVT1 shRNA (lentivirus-shPVT1) was purchased from GenePharma Biological Technology (Shanghai, China) to inhibit the expression of PVT1 in HCMs and HCM-derived exosomes. Lentivirus-shRNA control (shCtrl) was the negative control of lentivirus-shPVT1. Moreover, the PVT1 overexpression system (pcDNA-PVT1), miR-145-5p mimics, and IL-16 siRNA (si-IL-16) were transfected into THP-1 cells using Lipofectamine™ 2000 reagent (Invitrogen). In our study, pcDNA-PVT1 and its negative control, miR-145-5p mimics and mimics NC, and si-IL-16 were also purchased from GenePharma Biological Technology.

The gene sequences used in our study are as follows: miR-145-5p mimics: sense: 5′-GUCCAGUUUUCCCAGGAAUCCCU-3′, antisense: 5′-GGAUUCCUGGGAAAACUGGACUU-3′; mimics negative control: sense: 5′-UU CUCCGAACGUGUCACGUTT-3′, antisense: 5′-ACGUGACACGUUCGGAGAAT T-3′; IL-16 siRNA: sense: 5′-AGACAAAGUUCAAGUUCAACC-3′, antisense: 5′-UUGA ACUUGAACUUU GUCUUU-3′; and *PVT1* shRNA: 5′-CAGCCATCATGATGGTACT-3′.

### 2.8. Luciferase Activity Assay

To explore the combination among PVT1, miR-145-5p, and IL-16 3′-UTR, the binding sites between PVT1 and miR-145-5p and the binding sites between miR-145-5p and IL-16 3′-UTR were predicted using the starBase v2.0 database. Two different binding sequences between PVT1 and miR-145-5p were found. The wild-type (WT) or mutant (MUT) fragments of PVT1 and IL-16 3′-UTR, which contain the binding sites with miR-145-5p, were inserted into the luciferase reporter vector pGL3 (Promega, Fitchburg, WI, USA). The constructed plasmid of PVT1-WT, PVT1-MUT1, or PVT1-MUT2 was cotransfected with miR-145-5p mimics or mimics NC into THP-1 cells to examine the combination between PVT1 and miR-145-5p. Besides, the constructed plasmid of IL-16 3′-UTR-WT or IL-16 3′-UTR-MUT was cotransfected with miR-145-5p mimics, mimics NC, miR-145-5p inhibitor, or inhibitor NC into THP-1 cells to detect the combination between miR-145-5p and IL-16 mRNA.

### 2.9. RNA Immunoprecipitation (RIP) Assay

RIP assay was performed to explore whether PVT1 and miR-145-5p are together in the same RISC complex. In brief, THP-1 cells were maintained with RIPA lysis buffer to obtain cell lysates. Cell lysates were then incubated with human anti-Ago2 or anti-IgG (Millipore) overnight at 4°C. Then, the RNA-protein complexes were immunoprecipitated with protein A agarose beads. Next, the RNAs were isolated by using TRIzol reagent. Finally, the expression levels of miR-145-5p and PVT1 were measured by qRT-PCR assay. IgG served as the negative control.

### 2.10. Conditional Culture of Atrial Fibroblasts

Human atrial fibroblasts were isolated, identified and cultured as the previous study [12]. Human atrial fibroblasts were isolated from human atrial appendage tissues. The cells were cultured at 37°C in a humidified atmosphere containing 5% CO_2_ in DMEM medium supplemented with 20% FBS, 100 U/ml penicillin, 100 mg/ml streptomycin, and 250 ng/ml amphotericin B. Here, to explore the regulatory mechanism of HCM-derived exosomes in myocardial fibrosis, we collected the cell culture medium of THP-1. The exosomes derived from PBS-, Ang-II-, Ang-II+shCtrl-, or Ang-II+shPVT1-treated HCMs were collected. Next, the exosomes were incubated with THP-1 cells. 24 hours later, cell culture medium of THP-1 was renewed. Then, the supernatants were collected 12 hours later. THP-1 cell-conditional medium was used to culture atrial fibroblasts for 24 hours. At last, the expression of MMP-9, TIMP-1, collagen I, and collagen III in atrial fibroblasts was measured utilizing western blot assay. The protocol was approved by the Ethics Committee of the Renmin Hospital of Wuhan University. Informed written consent was obtained from all participants.

### 2.11. Statistical Analysis

Statistical analyses were performed using SPSS 20.0 software (SPSS, Inc., Chicago, IL, USA). The Student *t*-test was performed to analyze the difference between two independent groups, and one-way ANOVA followed by Tukey's post hoc test was carried out to ensure the difference among multiple groups. For all assays, the value of *P* lower than 0.05 was considered as statistically significant difference. All experiments were repeated three times at least.

## 3. Results

### 3.1. Ang-II-Treated HCM-Derived Exosomes Promoted M1 Macrophage Polarization

**Figure 1:**
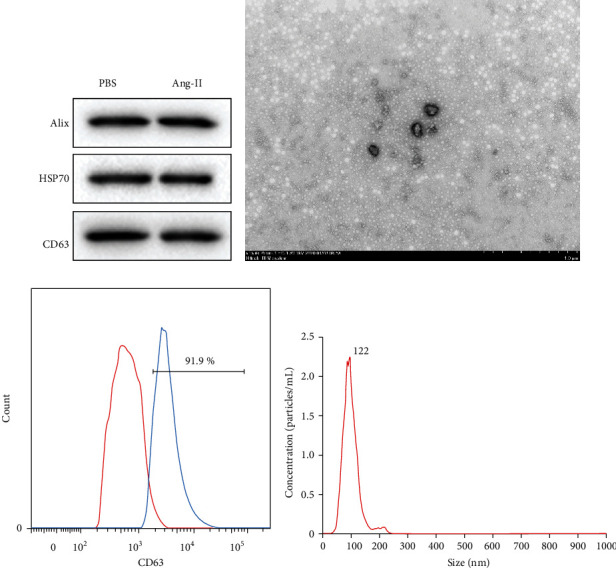
Identification of HCM-derived exosomes. (a) PBS or Ang-II-treated HCM-derived vesicles were collected. Then, the expression of specific markers of exosomes like Alix, HSP70, and CD63 was measured by western blot. (b) TEM was used to ensure the morphology of vesicles derived from Ang-II-treated HCMs. (c) Flow cytometry was performed to detect the percentage of CD63-positive vesicle derived from Ang-II-treated HCMs. (d) The average diameter of the Ang-II-treated HCM-derived vesicles was measured by NTA. *n* = 3.

In the beginning, we obtained the vesicles derived from HCM by density gradient centrifugation and verified the characteristics of exosome to ensure the follow-up experiments can be done successfully. Vesicles were collected from the culture medium of Ang-II- or PBS-treated HCM cells. Alix, HSP70, and CD63 are the characteristic surface markers of exosomes. Here, we determined the expression of Alix, HSP70, and CD63 in all vesicles (Figure 1(a)). Moreover, the typical cup-shaped morphology of exosomes was found in these vesicles using TEM (Figure 1(b)). The average diameter of exosome was 100 nm (Figure 1(d)). Besides, flow cytometry results showed that the percentage of CD63-positive vesicles was up to 91.9% (Figure 1(c)). Above data showed that we obtained PBS- or Ang-II-stimulated HCM-derived exosomes with 1 ~ 20 × 10^8^/ml concentration successfully.

**Figure 2:**
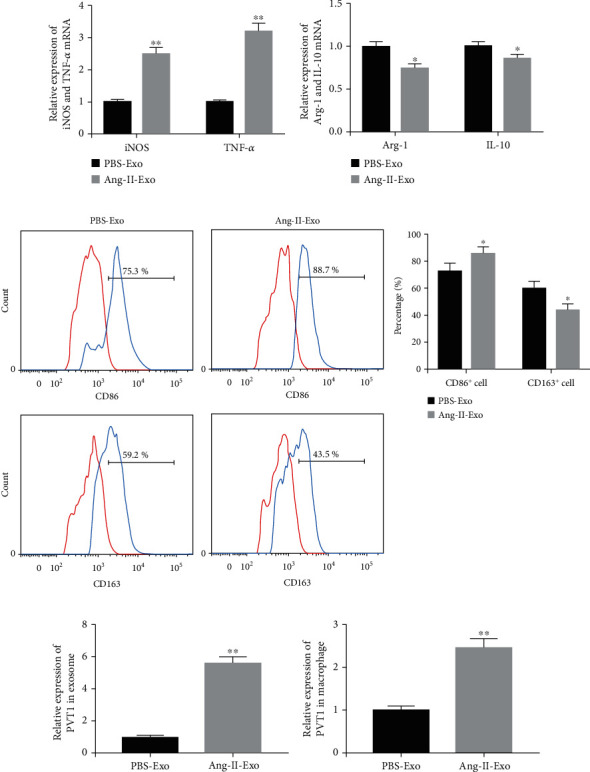
Ang-II-stimulated HCM-derived exosomes promoted M1 macrophage polarization. Exosomes were collected from the culture medium of HCM treated with PBS or Ang-II. Then, the exosomes were incubated with THP-1 cells. (a) qRT-PCR was carried out to detect the expression of iNOS and TNF-*α* mRNAs in THP-1 cells. (b) The expression levels of Arg-1 and IL-10 mRNAs were also measured by qRT-PCR. (c) The percentages of CD86-positive and CD163-positive macrophage were analyzed using flow cytometry. (d) The expression of PVT1 in PBS-Exo and Ang-II-Exo was measured by qRT-PCR. (e) The expression of PVT1 in macrophage was measured by qRT-PCR. PBS-Exo: PBS-treated HCM-derived exosomes; Ang-II-Exo: Ang-II-stimulated HCM-derived exosomes. *n* = 3. ^∗^*P* < 0.05 compared to the PBS-Exo group and ^∗∗^*P* < 0.01 compared to the PBS-Exo group. Student's *t*-test was performed to analyze the difference between two independent groups, and the value of *P* lower than 0.05 was considered as statistically significant difference.

Subsequently, the exosomes (80 *μ*g/ml) derived from PBS- and Ang-II-treated HCMs were added into the culture medium of THP-1 cells. At 24 hours later, the polarization of THP-1 cells was examined. Here, the qRT-PCR results showed that Ang-II-Exo could significantly increase the expression of iNOS and TNF-*α* mRNAs in macrophages (Figure 2(a)). TNF-*α* and iNOS are the representative indicators of M1-polarized macrophage. Oppositely, Ang-II-Exo reduced the expression of Arg-1 and IL-10 mRNAs in macrophages (Figure 2(b)). Arg-1 and IL-10 are the representative indicators of M2-polarized macrophage. In addition, CD86 and CD163 are the markers of M1 macrophage and M2 macrophage, respectively [15]. Here, we detected the percentages of CD86-positive and CD163-positive macrophage using flow cytometry. The results indicated that Ang-II-Exo promoted M1 macrophage polarization and limited M2 macrophage polarization (Figure 2(c)). Altogether, Ang-II-stimulated HCM-derived exosomes promoted macrophage to M1 polarization and suppressed M2 polarization.

### 3.2. Ang-II-Treated HCM-Derived Exosomes Facilitated M1 Macrophage Polarization via Submitting PVT1

**Figure 3:**
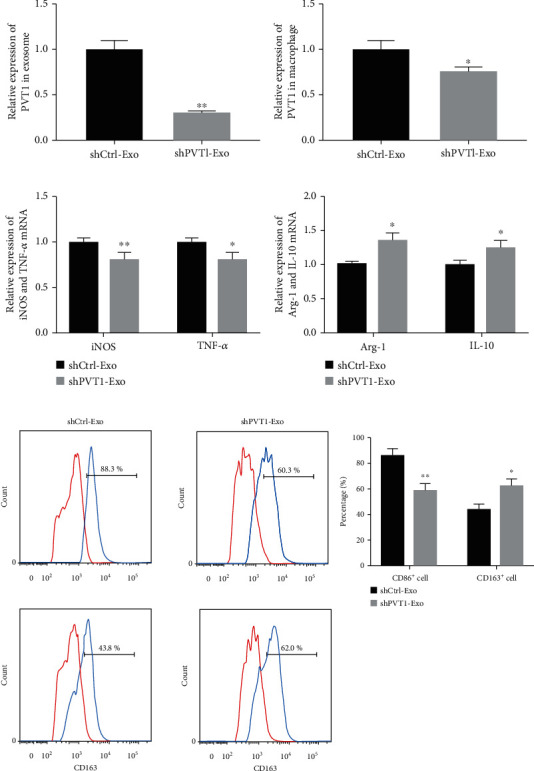
Ang-II-treated HCM promoted M1 macrophage polarization through secreting exosomal PVT1. Lentivirus expressing shPVT1 or shCtrl was infected with Ang-II-stimulated HCM for 24 hours. (a) Exosomes were collected from the culture medium of HCM, and then, the expression of PVT1 in exosomes was measured by qRT-PCR. (b) These two types of exosomes were incubated with THP-1 cells. The expression of PVT1 in THP-1 cells was measured using qRT-PCR. (c) The gene expression of iNOS and TNF-*α* was detected using qRT-PCR. (d) qRT-PCR was carried out to examine the expression of Arg-1 and IL-10 mRNAs. (e) Flow cytometry was performed to ensure the percentage of M1 and M2 macrophage. Here, shCtrl-Exo was lentivirus-shCtrl-infected HCM-derived exosomes and shPVT1-Exo was lentivirus-shPVT1-infected HCM-derived exosomes. *n* = 3. ^∗^*P* < 0.05 and ^∗∗^*P* < 0.01. Student's *t*-test was performed to analyze the difference between two independent groups, and the value of *P* lower than 0.05 was considered as statistically significant difference.

Our previous study demonstrated that PVT1 is upregulated in AF [12]. Here, our results indicated that the expression level of PVT1 also was upregulated in Ang-II-Exo (Figure 2(d)) as well as in Ang-II-Exo-treated macrophage (Figure 2(e)). Hence, we conjectured that the aberrantly expressed PVT1 may be involved in the regulation of HCM-derived exosomes to macrophage polarization. To identify whether HCM-derived exosomes regulate macrophage polarization through submitting PVT1, Ang-II-stimulated HCMs were infected with the lentivirus expressing shPVT1 or shCtrl. Our results showed that the expression level of PVT1 in both HCMs (Supplementary Figure 2) and shPVT1-Exo was downregulated by lentivirus-shPVT1 treatment (Figure 3(a)). Then, THP-1 cells were incubated with lentivirus-shCtrl- or lentivirus-shPVT1-treated HCM-derived exosomes. We used PKH67 to mark the lentivirus-shPVT1-treated HCM-derived exosomes and indicated that these exosomes can be effectively phagocytized by THP-1 cells (Supplementary Figure 1). The expression of PVT1 was significantly downregulated in the THP-1 cells treated with lentivirus-shPVT1-infected HCM-derived exosomes (Figure 3(b)). Subsequently, we further detected the polarization of macrophage. Our data suggested that decreasing the expression of PVT1 in Ang-II-Exo could inhibit the expression of M1 macrophage markers (Figure 3(c)), while facilitate the expression of M2 macrophage markers (Figure 3(d)). Furthermore, the results of flow cytometry also indicated that knockdown of PVT1 downregulated the percentage of M1 macrophage, but upregulated the percentage of M2 macrophage (Figure 3(e)). Overall, inhibition of PVT1 in Ang-II-stimulated HCMs accelerated M2 macrophage polarization. Ang-II-treated HCMs promoted macrophage to M1 polarization through submitting exosomal PVT1.

### 3.3. PVT1 Promoted the Expression of IL-16 through Acting as a miR-145-5p Sponge

**Figure 4:**
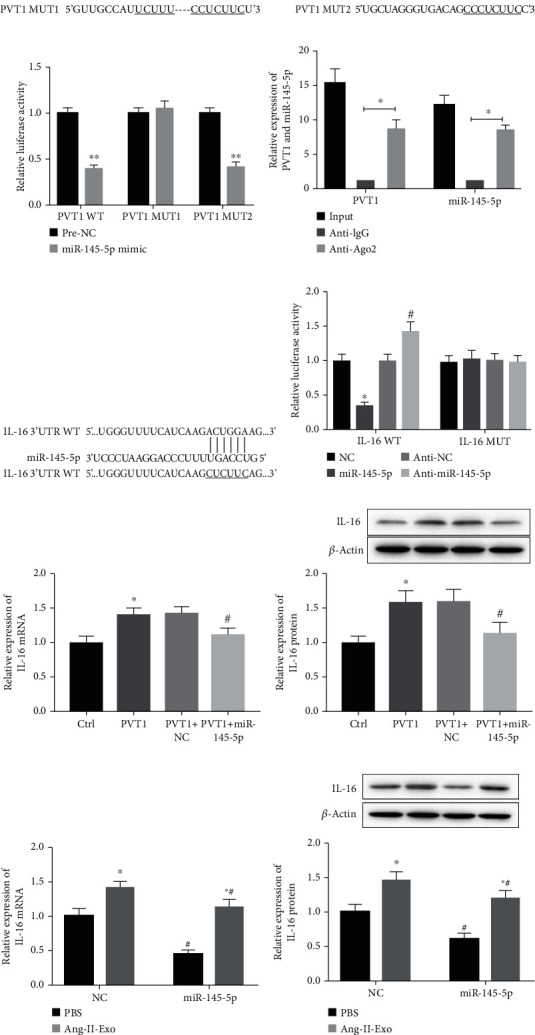
PVT1 promoted IL-16 expression through acting as a miR-145-5p sponge. (a) Two different binding sequences between PVT1 and miR-145-5p were predicted using starBase. (b) The relationship between PVT1 and miR-145-5p was identified using luciferase reporter assay. *n* = 3. ^∗^*P* < 0.05. (c) qRT-PCR analysis of PVT1 and miR-145-5p enrichment by Ago2 and IgG antibody in RIP experiments. *n* = 3. ^∗^*P* < 0.05. (d) The binding sites between miR-145-5p and IL-16 3′-UTR region were predicted using starBase. (e) Luciferase activity of THP-1 cotransfected with the reporter plasmid inserted WT or MUT IL-16 3′-UTR sequences and miR-145-5p mimics, mimics NC, miR-145-5p inhibitor, or inhibitor NC. *n* = 3. ^∗^*P* < 0.05. (f) The expression of IL-16 gene was measured by qRT-PCR. *n* = 3. ^∗^*P* < 0.05 compared with the Ctrl group and ^#^*P* < 0.05 compared with the PVT1 group. (g) The expression of IL-16 protein was determined using western blot. *n* = 3. ^∗^*P* < 0.05 compared with the Ctrl group and ^#^*P* < 0.05 compared with the PVT1 group. (h) qRT-PCR was performed to detect the expression of IL-16 gene. *n* = 3. ^∗^*P* < 0.05 compared with the PBS group, and ^#^*P* < 0.05 compared with the NC group. (i) Western blot was carried out to measure the expression of IL-16 protein. *n* = 3. ^∗^*P* < 0.05 compared with the PBS group, and ^#^*P* < 0.05 compared with the NC group. Student's *t*-test was performed to analyze the difference between two independent groups, and one-way ANOVA followed by Tukey's post hoc test was carried out to ensure the difference among multiple groups. For all assays, the value of *P* lower than 0.05 was considered as statistically significant difference.

To investigate the action mechanism of PVT1 to macrophage polarization, we verified the relationship among PVT1, miR-145-5p, and IL-16. Firstly, two different binding sequences between PVT1 and miR-145-5p were predicted using starBase v2.0 (Figure 4(a)). Then, the combination between PVT1 and miR-145-5p was examined using luciferase reporter assay. Our data revealed that PVT1 bound with miR-145-5p through the first binding sites (Figure 4(b)). Meanwhile, the combination between PVT1 and miR-145-5p was also proved by RIP assay. Our data showed that PVT1 and miR-145-5p were preferentially enriched in the anti-Ago2 group (Figure 4(c)). Subsequently, the binding sites between miR-145-5p and IL-16 3′-UTR were also predicted using the starBase v2.0 database (Figure 4(d)). The luciferase reporter assay proved that IL-16 3′-UTR acted as a downstream target of miR-145-5p (Figure 4(e)). To confirm whether PVT1 plays its function via promoting IL-16 expression by inhibiting miR-145-5p, we co-transfected pcDNA-PVT1 with miR-145-5p mimics into THP-1 cells. Our results revealed that increasing of PVT1 could notably promote the expression of IL-16 mRNA (Figure 4(f)) and protein (Figure 4(g)), while the promotory effect of PVT1 was partly reversed by miR-145-5p upregulation. Finally, we collected PBS-Exo and Ang-II-Exo to treat the THP-1 cells transfected with miR-145-5p mimics and mimics NC. As shown in Figures 4(h) and 4(i), Ang-II-Exo increased the expression of IL-16 mRNA and protein compared with the PBS-Exo-treated THP-1 cell. Both in the PBS-Exo- and Ang-II-Exo-treated THP-1, increasing of miR-145-5p suppressed IL-16 mRNA and protein expression. However, the enhancement of Ang-II-Exo to IL-16 expression also was effectively reversed by increasing in miR-145-5p. Taken together, exosomal PVT1 boosted the expression of IL-16 via acting as miR-145-5p sponge.

### 3.4. Exosomal PVT1 Promoted M1 Macrophage Polarization through Targeting the miR-145-5p/IL-16 Signaling Pathway

**Figure 5:**
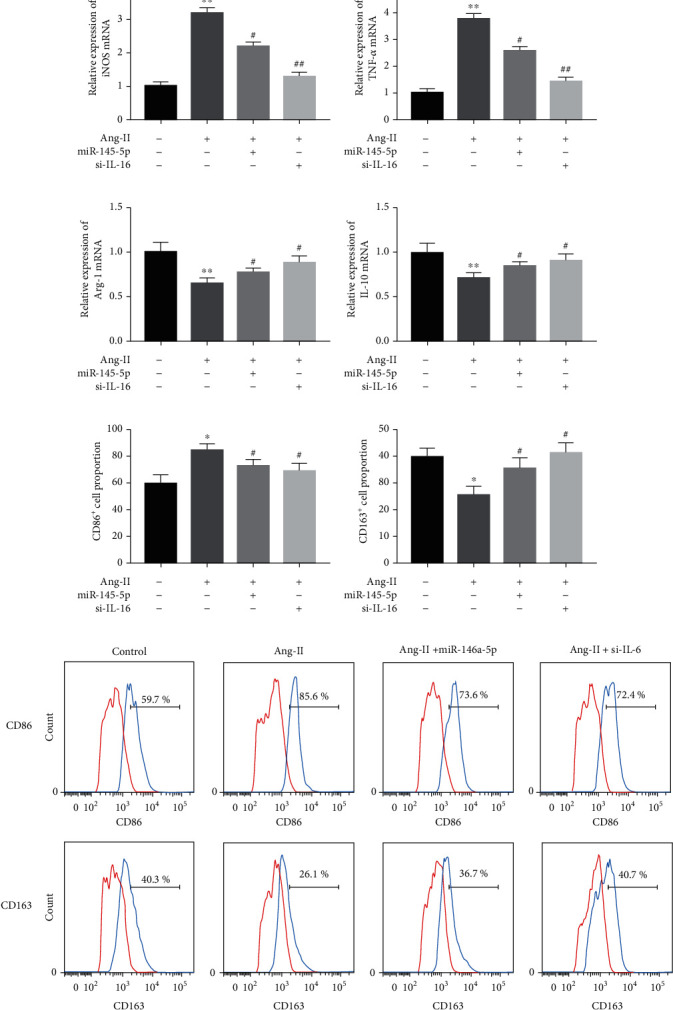
Ang-II-stimulated HCM-derived exosomes promoted M1 macrophage polarization via targeting the miR-145-5p/IL-16 signaling pathway. The expression of (a) iNOS mRNA, (b) TNF-*α* mRNA, (c) Arg-1 mRNA, and (d) IL-10 mRNA was measured by qRT-PCR. (e–g) The percentage of CD86-positive and CD163-positive THP-1 was detected using flow cytometry and analyzed. *n* = 3. ^∗^*P* < 0.05 and ^∗∗^*P* < 0.01 compared to the cells treated with nothing; ^#^*P* < 0.05 and ^##^*P* < 0.05 contrasted with the cells treated with Ang-II. One-way ANOVA followed by Tukey's post hoc test was carried out to ensure the difference among multiple groups. For all assays, the value of *P* lower than 0.05 was considered as statistically significant difference.

Then, we explored whether Ang-II-Exo promotes M1 macrophage polarization via upregulation of IL-16 by inhibiting miR-145-5p. Ang-II-Exo-treated THP-1 cells were transfected with miR-145-5p mimics or si-IL-16, and then, the polarization of macrophage was identified. Our data showed that both increasing the expression of miR-145-5p and decreasing the expression of IL-16 suppressed iNOS mRNA (Figure 5(a)) and TNF-*α* mRNA (Figure 5(b)) expression in Ang-II-Exo-treated THP-1 cells. Oppositely, both increasing miR-145-5p and decreasing of IL-16 reversed Ang-II-Exo-induced downregulation in the levels of Arg-1 mRNA (Figure 5(c)) and IL-10 mRNA (Figure 5(d)). The results of flow cytometry analysis indicated that the promotion of Ang-II-Exo to M1 macrophage polarization and inhibition to M2 macrophage polarization were recused by either miR-145-5p increasing or IL-16 decreasing (Figures 5(e)–5(g)). Overall, HCM-derived exosomes regulated the polarization of macrophage through targeting the miR-145-5p/IL-16 signaling pathway.

### 3.5. PVT1 Promoted the ECM Remodeling in Atrial Fibroblasts through Inducing M1 Macrophage Polarization

**Figure 6:**
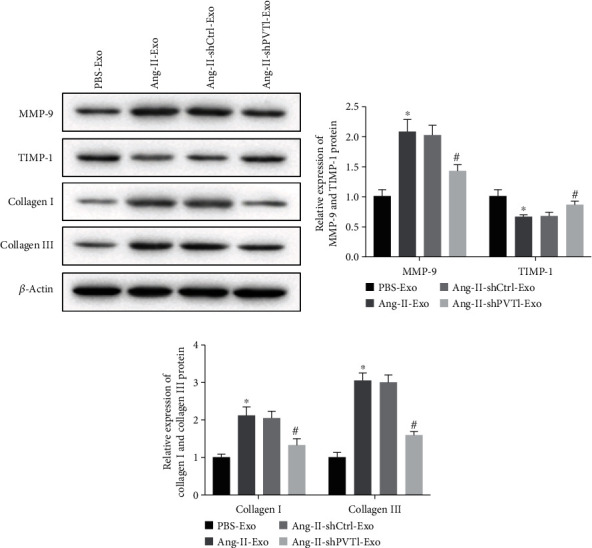
Ang-II-treated HCM-derived exosomes facilitated atrial fibrosis via targeting macrophage polarization. (a) The expression of MMP-9, TIMP-1, collagen I, and collagen III was measured by western blot. (b) Analysis of MMP-9 and TIMP-1 expression. (c) Analysis of collagen I and collagen III expression. *n* = 3. ^∗^*P* < 0.05 contrasted with the PBS-Exo group and ^#^*P* < 0.05 compared with the Ang-II-shCtrl-Exo group. One-way ANOVA followed by Tukey's post hoc test was carried out to ensure the difference among multiple groups. For all assays, the value of *P* lower than 0.05 was considered as statistically significant difference.

ECM remodeling and myocardial fibrosis play a crucial role in the development of AF. Here, to explore the regulatory mechanism of HCM-derived exosomes to myocardial fibrosis, the following experiments were done. THP-1 cells were treated with PBS-Exo, Ang-II-Exo, Ang-II+shCtrl-Exo, and Ang-II+shPVT1-Exo, and then, the cell culture medium of above medium was collected and was used for atrial fibroblast culture. Subsequently, the expression of MMP-9, TIMP-1, collagen I, and collagen III was measured using western blotting assay. As shown in Figures 6(a)–6(c), Ang-II-Exo-treated THP-1 cell-conditional medium upregulated the expression of MMP-9, collagen I, and collagen III, while it downregulated the expression of TIMP-1. However, above effects of Ang-II-Exo-treated THP-1 cell-conditional medium on MMP-9, collagen I, and collagen III expression in atrial fibroblasts were partly reversed by of PVT1 silencing. Meanwhile, the inhibition of Ang-II-Exo-treated THP-1 cell-conditional medium to TIMP-1 expression also was partly reversed by PVT1 silencing in HCMs. Overall, Ang-II-Exo promoted the ECM remodeling in atrial fibroblasts by regulating M1 macrophage polarization through submitting PVT1.

## 4. Discussion

AF is a serious global healthcare problem. Globally, the incidence and prevalence of AF are increasing continuously. The population of AF patients may be more than 50 million in 2030 [16]. Currently, although numerous drugs have already been used for the treatment of AF, such as antiarrhythmic drugs, *β*-blockers, and traditional Chinese medicine, these drugs have limited therapy outcomes. Existing drugs may increase the adverse events and mortality, and are not effective for some patients [17–19]. Hence, it is very necessary to explore the pathogenesis of AF and find a novel target for AF treatment. Electrical, structural, and contractile remodeling of the heart could accelerate AF development. ECM plays a crucial role in the remodeling [20]. Excessive ECM deposition or fibrosis occurs in almost all cardiac diseases [21]. It was known that ECM primary consists of collagens. Collagen I and collagen III are the main factors for the maintenance of structural integrity in myocardial tissues. In addition, collagens are considered as a predominant marker of fibrosis. During atrial fibrosis, the expression of collagen I and collagen III is increased in ECM [22]. Furthermore, MMPs and TIMPs (an inhibitor of MMPs) are also the important regulators in ECM remodeling. It was proved that the increasing of MMPs and downregulation of TIMPs lead to an enhancement of fibrosis [23, 24].

Exosome is a subtype of small vesicles enclosed by bilayer lipid membrane with a 40~100 nm diameter. Increasing evidence has demonstrated that exosomes are an important messenger among different cells via carrying lncRNA, miRNA, mRNA, and other molecules. Exosomes are involved in multiple biological processes and play a crucial role in the development of many disorders, including AF [25, 26]. However, the role of exosomes and exosomal lncRNAs in AF development remains not fully understood. In this study, we demonstrated that PVT1 is increased in Ang-II-Exo. Ang-II-Exo could promote macrophage to M1 polarization and inhibit M2 polarization by submitting PVT1. Interestingly, a previous study verified that PVT1 is also increased in the exosome derived from M2 macrophage in experimental autoimmune encephalomyelitis [27]. Macrophages are key inflammatory cells in AF development. It was indicated that both upregulation of the proportion of M1 macrophage and downregulation of M2 macrophage suppress atrial fibrosis and AF development. For instance, Sun et al. proved that lncRNA NRON attenuates AF via inhibiting atrial myocyte-activated M1 macrophage [28]. Here, we further indicated that inhibition of the expression of PVT1 in Ang-II-Exo suppresses ECM remodeling in atrial fibroblasts through promoting M2 macrophage polarization. Based on these findings, we thought that exosomal PVT1 may be a potential therapy target of AF.

According to competing endogenous RNA (ceRNA) mechanism, lncRNA could regulate the expression of mRNA via acting as miRNA sponge (endogenous inhibitors) [29]. Several studies have indicated that lncRNA can serve as ceRNA and play an important role in many disorders [30]. In our previous study, we revealed that PVT1 is increased in the atrial muscle tissues obtained from AF patients. Overexpression of PVT1 could promote Ang-II-induced atrial fibrosis through serving as miR-128-3p sponge. PVT1 accelerates the development of AF through promoting the expression of Sp1 via suppressing miR-128-3p expression [12]. In this study, we pointed out that PVT1 acts as a sponge of miR-145-5p. Importantly, exosomal PVT1 facilitated M1 macrophage polarization by increasing the expression of IL-16 via decreasing miR-145-5p expression. miR-145-5p is a common miRNA and is expressed in many cells like cancer cells and macrophage [31, 32]. The previous study demonstrated that miR-145-5p participates in the regulation of cell apoptosis and inflammatory response in hypoxia-induced cardiac myocytes [33].

## 5. Conclusions

In summary, our data demonstrated that Ang-II-treated HCM-derived exosomal PVT1 facilitates ECM remodeling in atrial fibroblasts through inducing macrophage to M1 polarization. Mechanistically, PVT1 promoted M1 macrophage polarization via increasing the expression of IL-16 by inhibiting miR-145-5p. Our study revealed a new regulatory mechanism of atrial fibroblasts in AF as well as pointed out a novel evidence to support exosomal PVT1 acting as the potential target for AF treatment. Nevertheless, there is still a lot of work that needs to be done. For instance, our results were not measured *in vivo*. Whether the regulatory mechanism of Ang-II-treated HCM-derived exosomal PVT1 in AF also applies to animal experiments is unclear and whether an inhibitor of PVT1 could effectively improve AF. Besides, how about the expression of PVT1 in macrophage-derived exosome in AF? We will perform more work to improve our study.

## Data Availability

The datasets and codes generated or analyzed in this study are available from the corresponding author upon reasonable request.

## Supplementary Materials

Supplementary 1 Supplementary Figure 1: Ang-II-Exo was phagocytized by THP-1 cells. Ang-II-treated HCM-derived exosomes were labeled with PKH67 and then were incubated with THP-1 cells. The distribution of exosomes in THP-1 cells was observed using a confocal microscope.

Supplementary 2 Supplementary Figure 2: detection of the infection efficiency of shPVT1. HCMs were treated with the lentivirus expressing PVT1 shRNA-1, PVT1 shRNA-2, or shRNA negative control. Then, the expression of PVT1 in HCMs was measured by qRT-PCR. Student's *t*-test was performed to analyze the difference between two independent groups, and the value of *P* lower than 0.05 was considered as statistically significant difference.

Supplementary 3 Supplementary Table 1: the primer sequences of genes used in qRT-PCR assay.
