# Supplementary figures and images for: Angiotensin II-Treated Cardiac Myocytes Regulate M1 Macrophage Polarization via Transferring Exosomal PVT1
Source: J Immunol Res. 2021 Aug 30;2021:1994328. doi: 10.1155/2021/1994328 (PMC8427676; doi:10.1155/2021/1994328)

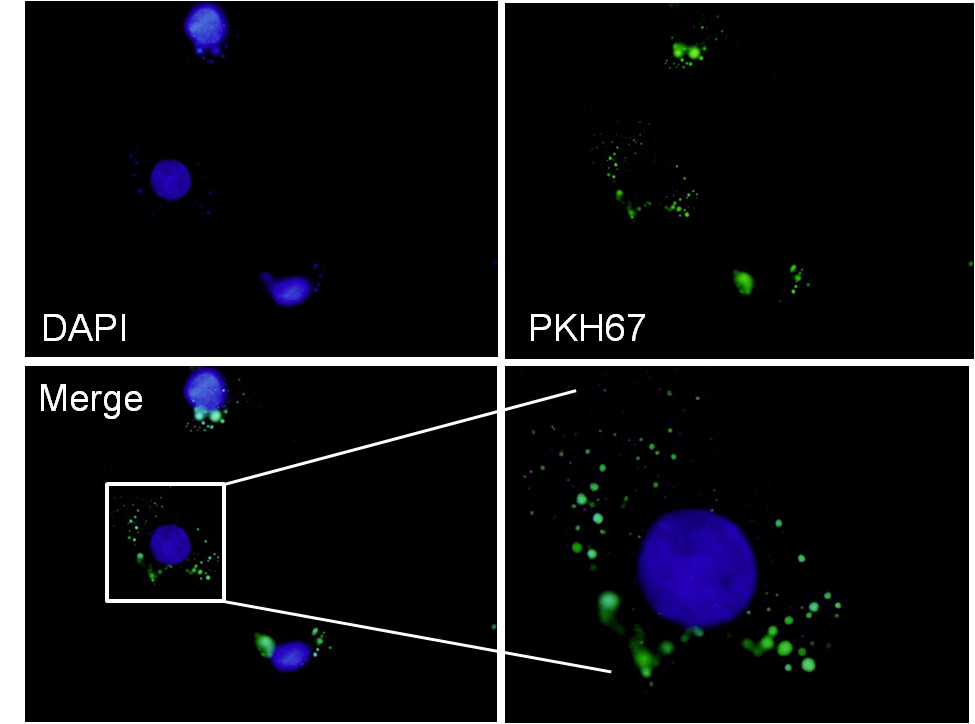

Supplement: Supplementary 1 — Supplementary Figure 1: Ang-II-Exo was phagocytized by THP-1 cells. Ang-II-treated HCM-derived exosomes were labeled with PKH67 and then were incubated with THP-1 cells. The distribution of exosomes in THP-1 cells was observed using a confocal microscope. [file 1994328.f1.jpg]

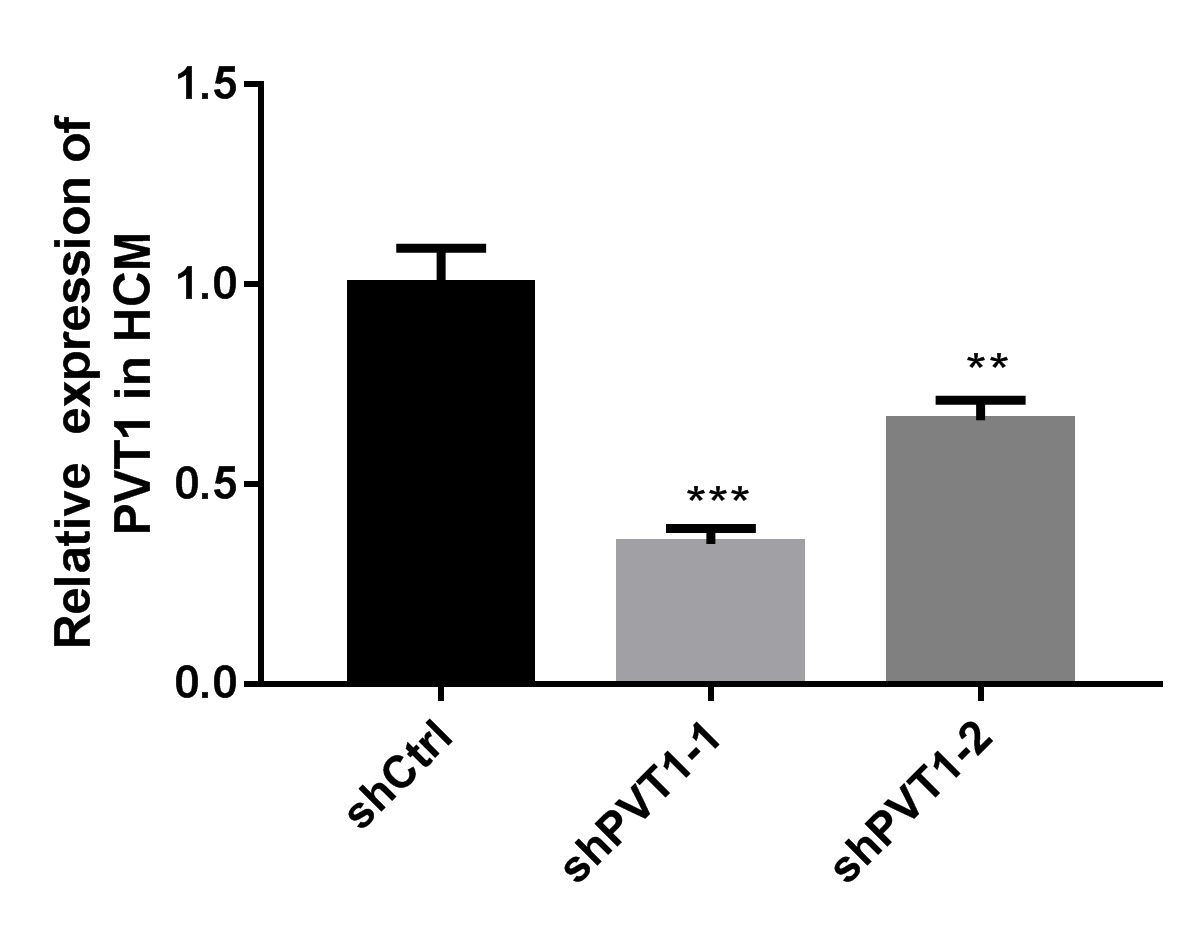

Supplement: Supplementary 2 — Supplementary Figure 2: detection of the infection efficiency of shPVT1. HCMs were treated with the lentivirus expressing PVT1 shRNA-1, PVT1 shRNA-2, or shRNA negative control. Then, the expression of PVT1 in HCMs was measured by qRT-PCR. Student's t-test was performed to analyze the difference between two independent groups, and the value of P lower than 0.05 was considered as statistically significant difference. [file 1994328.f2.jpg]
