# Supplementary material for: Angiotensin II-Treated Cardiac Myocytes Regulate M1 Macrophage Polarization via Transferring Exosomal PVT1
Source: J Immunol Res. 2021 Aug 30;2021:1994328. doi: 10.1155/2021/1994328 (PMC8427676; doi:10.1155/2021/1994328)
Supplement: Supplementary 3 — Supplementary Table 1: the primer sequences of genes used in qRT-PCR assay. [file 1994328.f3.docx]

| Gene name | Forward (5’-3’) | Reverse (5’-3’) |
| --- | --- | --- |
| TNF-α | 5’-CCACCACGCTCTTCTGTC-3’ | 5’-ATCTGAGTGTGAGGGTCTGG-3’ |
| iNOS | 5’-TGGCTGTGGTCACCTATCG-3’ | 5’-GGTCTTCGGGCTTCAGGTTA-3’ |
| Arg-1 | 5’-TGCAGTGGCAGAAATCAAGA-3’ | 5’-AGCATCCACCCAAATGACA-3’ |
| IL-10 | 5’-GGCTCAGCACTGCTATGTTGCC-3’ | 5’-AGCATGTGGGTCTGGCTGACTG-3’ |
| PVT1 | 5’-TGAGAACTGTCCTTACGTGACC-3’ | 5’-AGAGCACCAAGACTGGCTCT-3’ |
| IL-16 | 5’-ATGCCCGACCTCAACTCC-3’ | 5’-CTAGGAGTCTCCAGCAGC−3’ |
| miR-145-5p | 5’-CGGTCCAGTTTTCCCAGGAA-3’ | 5’-AGTGCAGGGTCCGAGGTATT-3’ |
| GAPDH | 5’-TGTTCGTCATGGGTGTGAAC-3’ | 5’-ATGGCATGGACTGTGGTCAT-3’ |
| U6 | 5’-CTCGCTTCGGCAGCACA-3’ | 5’-AACGCTTCACGAATTTGCGT-3’ |
